# Expression, purification, electron microscopy, N-glycosylation mutagenesis and molecular modeling of human P2X4 and *Dictyostelium discoideum* P2XA

**DOI:** 10.1016/j.bbamem.2011.08.025

**Published:** 2011-12

**Authors:** Maria Valente, Summer J. Watterson, Mark D. Parker, Robert C. Ford, Mark T. Young

**Affiliations:** a School of Biosciences, Cardiff University, Museum Avenue, Cardiff, CF10 3AX, UK; b Department of Physiology and Biophysics, Case Western Reserve University School of Medicine, 10900 Euclid Ave, Cleveland, OH, USA; c Manchester Interdisciplinary Biocentre, University of Manchester, 131 Princess Street, Manchester, M1 7DN, UK

**Keywords:** β-OG, n-octyl-β-d-glucoside, C_12_E_8_, octaethylene glycol monododecyl ether, DDM, n-dodecyl-β-d-maltoside, FC-12, n-dodecylphosphocholine, LDAO, n-dodecyl-N,N-dimethylamine-N-oxide, PBS, phosphate-buffered saline, SDS, sodium dodecyl sulfate, Sf9 cells, *Spodoptera frugiperda* cell-line derived from pupal ovaries, TEM, transmission electron microscopy, TX-100, Triton-X-100, zf, zebrafish, P2X, Sf9, TEM, Single particle analysis, N-glycosylation, Modeller

## Abstract

The recent publication of the *apo-*, closed-state 3D crystal structure of zebrafish (zf) P2X4.1 has not only revolutionized the P2X research field, but also highlighted the need for further crystal structures, of receptors in different activation states, so that we can gain a complete molecular understanding of ion channel function. zfP2X4.1 was selected as a 3D-crystallization candidate because of its ability to form stable trimers in detergent solution, and purified from over-expression in baculovirus-infected *Spodoptera frugiperda* (Sf9) insect cells. In this work, we have used a similar approach to express both human P2X4 (hP2X4) and *Dictyostelium discoideum* P2XA (DdP2XA) in Sf9 cells. Although hP2X4 did not form stable trimers in detergent solution, both receptors bound to ATP-coupled resins, indicating that their extracellular domains were folded correctly. DdP2XA formed strong trimers in detergent solution, and we were able to selectively purify trimers using preparative electrophoresis, and build a 21 Å-resolution 3D structure using transmission electron microscopy and single particle analysis. Although the structure of DdP2XA possessed similar dimensions to those of the previously determined low-resolution hP2X4 structure and the zfP2X4.1 crystal structure, N-glycosylation mutagenesis and molecular modeling indicated differences between N-glycan usage and predicted accessibility in models of DdP2XA based on the zfP2X4.1 crystal structure. Our data demonstrate that DdP2XA expressed in insect cells retains ATP-binding capacity after detergent solubilization, is an ideal candidate for structural study, and possesses a significantly different 3D structure to that of both hP2X4 and zfP2X4.1.

## Highlights

► We expressed human P2X4 and *Dictyostelium discoideum* (Dd) P2XA in Sf9 insect cells. ► Both receptors were capable of binding to ATP-coupled sepharose beads. ► Preparative electrophoresis was used to purify DdP2XA trimers. ► We determined a 21 Å-resolution structure of DdP2XA using single particle analysis. ► DdP2XA cannot be modeled accurately using the zebrafish P2X4 crystal structure.

## 1 Introduction

P2X receptors are trimeric cation channels gated by extracellular ATP. There are seven subtypes in mammals, which display differential agonist/antagonist selectivity and ion channel properties [1], and play roles in physiological processes such as nerve transmission, pain sensation, control of vascular tone and inflammation, making them key drug targets [2,3]. In addition, P2X receptors are found in zebrafish (zf) [4], and several lower eukaryotes including the slime mold *Dictyostelium discoideum*[5]. The publication in 2009 of the first crystal structure of a P2X receptor, *apo-*zfP2X4.1 at 3.1 Å resolution [6] represents a major advance. Success with zfP2X4.1 was dependent upon three main factors: (i) screening of many different receptor constructs to find those which formed the most stable trimers in detergent solution [7]; (ii) use of the Sf9 insect cell expression system; and (iii) significant construct modification to achieve well-diffracting crystals. The extensive construct modification altered ion channel function; peak currents were reduced, channel closure after removal of agonist was delayed, and agonist potency was increased [6], implying that the structure may not represent a native conformation, but rather an artificially stabilized, closed state. Nevertheless, it has enabled the interpretation of a large quantity of previous experimental data relating to transmembrane domain orientation, ligand-binding and channel opening in molecular detail [8–10], and serves as an excellent template from which to model other P2X receptors.

To fully understand the molecular basis of agonist/antagonist binding, channel activation and subtype selectivity, further P2X receptor structures, including the ligand-bound state (in closed and open conformation), and structures of different receptor subtypes (particularly the mammalian receptors) are needed. Obtaining these structures represents a huge challenge because it is difficult to produce stable, folded P2X receptors in the milligram-quantities required for 3D crystal trials. Thus far, successful over-expression of P2X receptors for structural study has been achieved in mammalian cell lines for rat P2X2, rat P2X6 [11] and human P2X4 [12,13], and in baculovirus-infected Sf9 insect cells for rat P2X2 [14,15] and zfP2X4.1 [6]. However, in most cases, the yield of protein has been too low to permit 3D crystallography, and structural data have been limited to low-resolution studies using atomic force microscopy [11,12,15] and single particle analysis [13,14,16], which require significantly lower quantities of protein. By far the most successful approach has been that of Kawate et al., determining the construct most likely to produce a high yield of stable trimers, and expressing this in Sf9 cells [6].

To exploit and extend this recent advance, we have employed the Sf9 insect cell system to express both human P2X4 (hP2X4) and *D. discoideum* P2XA (DdP2XA). hP2X4 is the nearest human homologue to zfP2X4.1, sharing 57% amino acid identity. It has also been shown to form a significant proportion of stable trimers when over-expressed in human cells [13]. DdP2XA is more distantly related to zfP2X4.1, sharing only 16% amino acid identity. However, this protein has been shown to form highly stable trimers when expressed in human cells [5], which was an important indicator of success with zfP2X4.1 [6].

We assessed the degree of trimer formation of each receptor in a variety of detergents using non-denaturing perfluorooctanoic acid (PFO)–PAGE, and found that while hP2X4 did not form stable trimers in any of the detergents tested, DdP2XA was very stable in several detergents, including n-dodecyl-β-d-maltoside (DDM). We also assessed whether or not the receptors were folded by performing pull-down assays with ATP-coupled sepharose beads; both receptors bound to beads coupled to ATP at the γ-phosphate- or 6-amino-position, but not at the 8-position, which may give some indication of how ATP is positioned within the binding site. Following selective purification of DdP2XA trimers using preparative PFO–PAGE, electron microscopy and single particle analysis were used to build a low-resolution 3D structure of the receptor, which possessed pronounced extracellular-domain propellers compared to the previously determined hP2X4. Finally, we generated molecular models of both hP2X4 and DdP2XA using the zfP2X4.1 crystal structure as the template, and probed their accuracy using N-glycosylation mutagenesis, finding several discrepancies between actual N-glycan usage in DdP2XA and predicted residue accessibility.

Our data show that (i) wild-type hP2X4 expressed in insect cells does not form stable trimers in detergent solution, but is still capable of binding ATP; and (ii) wild-type DdP2XA forms stable trimers in detergent, is an excellent candidate for over-expression in Sf9 cells for structure determination, and that its overall 3D-structure appears to be significantly different to that predicted from molecular modeling studies.

## 2 Experimental

### 2.1 cDNA constructs, mutagenesis, expression in HEK cells and Western blotting

cDNA constructs corresponding to human P2X4-(His)_10_ and human codon-optimized DdP2XA-(His)_6_ have been described previously [5,13]. Site-directed mutagenesis was carried out using the QuikChange kit (Stratagene) according to manufacturers' instructions. HEK-293 cells were cultured to approximately 80% confluency in 35-mm dishes, and transfected with 1 μg cDNA using 3 μl Lipofectamine (Invitrogen) according to manufacturers' instructions. 24 hours post-transfection, cells were washed twice in ice-cold phosphate-buffered saline (PBS, Sigma) and pelleted by centrifugation at 4000*g* for 3 minutes. Total protein samples were prepared by solubilizing the cells from one 35-mm dish in 50 μl PBS containing 1% (w/v) n-dodecyl-β-d-maltoside (DDM) and protease inhibitors (Roche Complete—EDTA). Solubilized protein concentrations were determined using Bradford assay, protein samples were denatured by boiling for 2 minutes at 100 °C, separated by SDS–PAGE on 10% polyacrylamide gels (Bio-Rad) and transferred to PVDF membranes. Membranes were blocked with 3% bovine serum albumin (BSA) for 1 hour at room temperature and incubated in blocking buffer with mouse monoclonal anti-His primary antibody (Qiagen; 1:2000 dilution) overnight at 4 °C. After washing in 3 changes of PBS containing 0.1% Tween-20 (PBS-T, Bio-Rad), membranes were incubated with either alkaline phosphatase- or HRP-coupled secondary antibody (Sigma or DAKO Cytomation; 1:2000) for 1 hour at room temperature and washed 4× in PBS-T. Blots were developed using either BCIP/NBT tablets (Sigma) or the ECL-Plus kit (GE Healthcare) according to manufacturers' instructions.

### 2.2 Generating recombinant bacmid for Sf9 expression

Our starting point was the above-mentioned clones which were used as templates for PCR. cDNA encoding human P2X4-(His)_10_ and human codon-optimized DdP2XA-(His)_6_ was amplified from these templates to include a CACC sequence in place of the initiation codon at the 5′ end and a termination codon at the 3′ end. The PCR products were subcloned into an insect cell expression vector using the Gateway system (Invitrogen, Carlsbad, CA). Briefly, cDNAs were subcloned into the “entry vector” pENTR–D-TOPO and recombined into the “destination vector” pDEST8 according to the manufacturer's recommendations. The sequence of pDEST8 clones was confirmed by the Keck DNA sequencing facility (New Haven, CT). pDEST8 clones were transformed into DH10Bac cells (Invitrogen) which generated recombinant bacmid that was isolated and purified from the DH10Bac cells according to manufacturer's recommendations. The presence of P2X cDNA in the bacmid was confirmed by PCR. 1 μg of the recombinant bacmid DNA was used to transfect Sf9 cells.

### 2.3 Culture, transfection, and harvesting of Sf9 cells

Serum-free medium (SFM) adapted Sf9 cells (Invitrogen) were seeded at a density of 9 × 10^5^ cells per well in a 6-well plate that contained 2 ml of Sf-900 II SFM (Invitrogen). Cells were allowed to attach for 1 hour at 27 °C, the medium was removed and cells were transfected by drop-wise addition of transfection mixture (1 μg bacmid, 6 μl cellfectin, 1 ml Sf-900 II SFM). Cells were incubated with the transfection mixture for 5 hours at 27 °C, after which time the transfection mixture was aspirated and replaced with 2 ml of Sf-900 II SFM. Transfected cells were incubated for 3 days at 27 °C and the supernatant (presumed to contain a low-titer of baculovirus particles) was harvested. 60 μl of the supernatant was used to infect further insect cells in a 6-well plate to produce 2 ml of high-titer viral stock. The viral titer was determined to be 2 × 10^7^ using the BacPAK baculovirus rapid titer kit (Clontech, Mountain View, CA) and expression of P2X in these cells was confirmed by Western blotting using an anti-His antibody (GE Healthcare, Waukesha, WI). This high-titer viral stock was used to infect progressively larger volumes of Sf9 cells that were cultured in suspension in ProCulture spinner flasks (Corning Inc., Corning, NY), which in turn generated progressively larger volumes of high-titer viral stock. Finally, 1 L of Sf9 cells grown in suspension culture to a density of 1 × 10^6^ cells/ml in Sf-900 II SFM were infected with viral particles at a multiplicity of infection of 0.5 (viral particles per cell). After 3 days, we harvested cell pellets by centrifugation at 500*g* for 15 minutes. Cell pellets were stored at − 80 °C.

### 2.4 Sf9 cell membrane preparation, detergent screening and PFO–PAGE

**Fig. 1:**
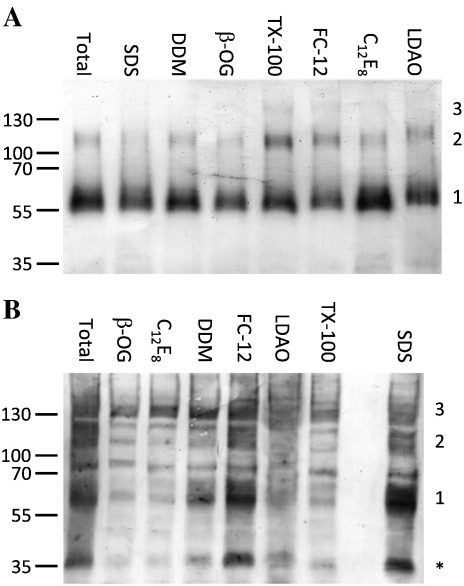
Detergent screening of hP2X4 and DdP2XA. Western blots (anti-His tag) of 10 μg membrane protein samples of hP2X4 (A) or DdP2XA (B) solubilized in a 1% (w/v) solution of each detergent indicated and separated using PFO–PAGE. Total, membrane suspension; SDS, sodium dodecyl sulfate; DDM, n-dodecyl-β-d-maltoside; β-OG, n-octyl-β-d-glucoside; TX-100, Triton-X-100; FC-12, n-dodecylphosphocholine; C_12_E_8_, octaethylene glycol monododecyl ether; LDAO, n-dodecyl-N,N-dimethylamine-N-oxide. Bands corresponding to monomers (1), dimers (2) and trimers (3) are indicated. Markers in kDa. *, partial degradation of DdP2XA.

All steps were performed on ice and protease inhibitors were present throughout. Sf9 cells from a 5-litre culture were resuspended in 50 ml phosphate-buffered saline (PBS) and disrupted by sonication for 6 × 5 seconds at 40% amplitude, with 10 seconds rest between pulses. Lysis was judged to be complete by light microscopy. Cell lysates were centrifuged at 2000*g* for 2 × 5 minutes to pellet cell debris. Membranes were recovered from the supernatant by centrifugation at 100,000*g* for 40 minutes, washed once with PBS pH 9.0 and once with PBS containing 500 mM NaCl and resuspended in PBS. Samples of insect cell membranes corresponding to cells from approximately 10 ml total culture volume were solubilized in TBS containing 1% (w/v) detergent (see Fig. 1 for detergents used) for 1 hour on ice, and centrifuged at 100,000*g* for 1 hour to pellet non-solubilized material. Equal volumes of supernatant (containing solubilized membrane protein) were mixed 1:1 with perfluoro-octanoic acid (PFO)–PAGE loading buffer (final concentration of 4% (w/v) PFO) [17], separated on 6% (w/v) gels and subjected to Western blotting.

### 2.5 Pull-down assays with ATP-coupled sepharose beads

100 μg samples of protein solubilized in PBS containing 1% (w/v) n-dodecyl-β-d-maltoside (DDM) were incubated with 20 μl of either γ-amino-hexyl ATP-, 8-amino-hexyl ATP- or N^6^-(4-amino)-hexyl ATP-sepharose beads (Jena Bioscience) for 1 hour at room temperature. Samples were centrifuged at 5000*g* for 1 minute to pellet beads, and the supernatant was removed (Flow fraction). Beads were washed with 3 × 0.5 ml of PBS containing 0.1% DDM, and protein was eluted by incubation with 100 μl 10 mM ATP (in PBS containing 0.1% DDM) for 5 minutes. The elution fraction was removed, and 100 μl SDS–PAGE sample buffer was added to the beads, which were boiled for 5 minutes at 100 °C to elute bound protein still bound to the beads. 10 μl samples of each fraction were separated by SDS–PAGE and subjected to Western blotting.

### 2.6 Protein purification

DDM-solubilized Sf9 cell membranes from a 5-litre culture were mixed 1:1 with wash buffer (Tris-buffered saline (20 mM Tris–HCl pH 7.9, 150 mM NaCl) containing 25 mM imidazole and 0.05% DDM) and 500 μl Ni-sepharose beads (GE healthcare) and incubated overnight at 4 °C to bind DdP2XA-(His)_6_ protein. The mixture was poured into an empty PD-10 column (with bottom frit; GE healthcare) and the flow-through fraction was allowed to pass through. A second frit was added above the beads to prevent drying. Beads were washed with 20 ml wash buffer and DdP2XA was eluted in a step-wise fashion using 2 × 0.5 ml aliquots of wash buffer containing 100, 200, 300, 400 and 500 mM imidazole. Samples were separated by SDS–PAGE and gels were stained with InstantBlue (Expedeon) to detect protein. To selectively purify DdP2XA trimers, elutions containing DdP2XA were pooled and concentrated to a volume of 1 ml in 100-kDa cut-off Vivaspin-6 centrifugal concentrators (Generon). The pooled part-purified DdP2XA was then mixed 1:1 with PFO–PAGE sample buffer and applied to an 8% (w/v) polyacrylamide tube gel in a cooled Bio-Rad 491 prep-cell in a cold room (4 °C). The gel was run at 12 W constant power, and 1 ml elutions were collected at a flow rate of 0.1 ml/minute once the dye front reached the bottom of the gel. Thirty fractions in total were collected; these were pooled into 6 × 5 ml fractions and concentrated to approximately 1 ml in 100-kDa cut-off Vivaspin-6 centrifugal concentrators. Samples of each fraction were subjected to PFO–PAGE and gels were stained with InstantBlue to determine the location of DdP2XA trimers within the fractions.

### 2.7 Transmission electron microscopy and single particle analysis

Protein samples (50 μg/ml in Tris-buffered saline containing 0.05% DDM) were adsorbed on to glow-discharged carbon-coated copper grids and negatively stained with 2% (w/v) uranyl acetate. Transmission electron microscopy images were recorded at a specimen level increment of 3.30 Å/pixel using a Jeol JEM-1200 electron microscope operating at 100 kV, equipped with a 2 k Gatan Orius camera, at the University of Manchester. EMAN software [18] was used to manually select, filter, and process 5021 particles in 64 × 64 pixel boxes. The final model was generated from a total of 32 iterations of refinement (the final 8 iterations with un-filtered particles) and Fourier shell correlation (FSC) analysis of structures generated from even-and odd-numbered particles using the command “eotest” indicated a resolution of ~ 21 Å. Structures were displayed at volume shells corresponding to at least 3 S.D. values above the mean density using UCSF Chimera [19].

### 2.8 Homology modeling

Sequences of 51 full-length P2X receptors were aligned using ClustalW (EBI) to obtain an optimal alignment within the boundaries of the zfP2X4.1 sequence contained within the crystal structure. Pairwise alignments of zfP2X4.1 and either hP2X4 or DdP2XA were prepared from the full alignment for use in the program Modeller [20]. In addition, a manual pairwise alignment of zfP2X4.1 and DdP2XA was prepared where DdP2XA lacks the majority of the cysteine-rich head domain (Supp. Fig. S1; based on an alignment published by Surprenant and North [3]). A trimer structure of zfP2X4.1, prepared from PDB code 3H9V, was used as the template, and 5 models were prepared using the standard script (model-default.py). Each model was assessed for quality using MolProbity [21] and the best model was subjected to energy minimization in UCSF Chimera [19]. This step further improved the quality of the models (as measured by MolProbity).

## 3 Results

### 3.1 Expression and trimer formation of hP2X4 and DdP2XA

To measure the degree of trimer formation after solubilization in detergent, aliquots of resuspended membranes corresponding to 10 ml original culture (0.2% total volume) were used in detergent screening experiments (Fig. 1). Samples were subjected to PFO–PAGE followed by Western blotting to detect His-tagged protein. PFO is a much milder detergent than SDS; it coats proteins with negative charge but does not denature them. It has been used previously to analyze the oligomeric state of a number of different membrane proteins [17], as well as hP2X4 and DdP2XA expressed in human cells [5,13]. Both untreated (total) and SDS-solubilized membranes (SDS) were included as controls. Monomeric hP2X4 displayed a molecular mass of approximately 55 kDa on PFO–PAGE gels (Fig. 1A); bands of 110 kDa (corresponding to dimer) were most prominent in samples treated with no detergent (Total), DDM, Triton X-100 (TX-100) and n-dodecylphosphocholine (FC-12). Bands of 165 kDa (trimers) were very faint in TX-100 and virtually absent in all other samples, including untreated membranes, leading to the conclusion that hP2X4 expressed in insect cells does not form stable trimers. No dimer or trimer formation was observed in SDS. Monomeric DdP2XA also displayed a mass of approximately 55 kDa on PFO–PAGE gels (Fig. 1B; SDS-treated lane). In addition, a band at 35 kDa was also observed in some samples (Fig. 1B; *); this probably represents a degradation product of the full-length receptor. The untreated membrane fraction was a smear of several bands and was difficult to interpret, but in the detergent-treated samples, bands corresponding to dimers and trimers were observed (Fig. 1B). The trimer bands were the major species in n-octyl-β-d-glucoside (β-OG), octaethylene glycol monododecyl ether (C_12_E_8_) and DDM, leading to the conclusion that DdP2XA formed stable trimers in a variety of detergents. A small proportion of trimer formation was observed in SDS, which may indicate the inherent stability of the receptor, or a proportion of misfolded protein which is aggregating. However, no higher molecular mass species than trimers were observed in SDS, which would argue against non-specific protein aggregation.

### 3.2 Expressed protein binds to ATP-coupled sepharose beads

**Fig. 2:**
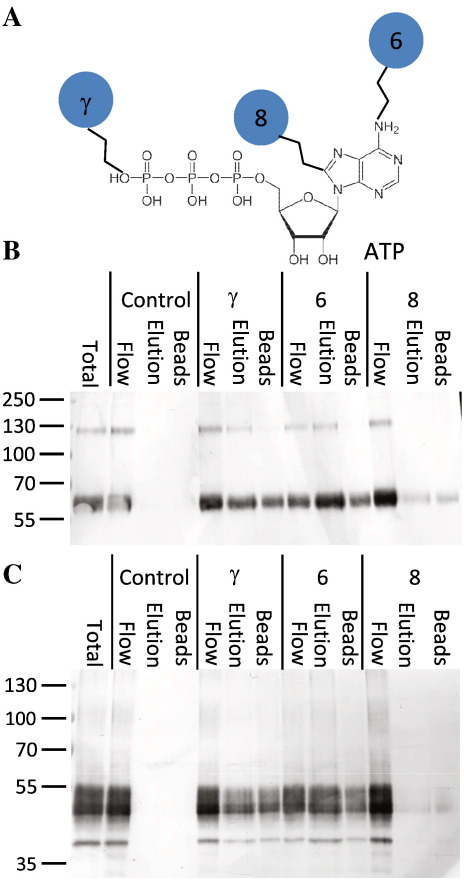
Binding of hP2X4 and DdP2XA to ATP-coupled sepharose beads. A: Chemical structure of ATP showing coupling to sepharose beads at the γ-, 6-amino- or 8-positions. B, C: Western blots (anti-His tag) of equal volumes of DDM-solubilized membrane protein samples of hP2X4 (B) or DdP2XA (C) before and after incubation with sepharose beads (control), γ-ATP coupled beads (γ), 6-amino-ATP coupled beads (6) or 8-ATP coupled beads (8). Flow, unbound protein; Elution, protein released by incubation with 10 mM ATP; Bound, protein remaining on the beads after elution. Markers in kDa.

Our initial intention was to trial a number of different ATP-coupled resins for use as a second affinity-purification step. To this end, we incubated DDM-solubilized membranes with sepharose beads coupled to ATP at the γ- 6-amino or 8-position in batch phase (Fig. 2), and eluted protein with 10 mM ATP. Blank sepharose beads were used as a negative control, and samples of beads were boiled in SDS–PAGE sample buffer to check for bound protein. Both hP2X4 (Fig. 2B) and DdP2XA (Fig. 2C) bound to beads coupled at the γ- and 6-amino positions, and a proportion of protein could be eluted with 10 mM ATP. However, a significant amount of P2X receptor did not bind (Flow), and also, a significant amount of the receptor that did bind could not be eluted from the beads with 10 mM ATP (Bound). Neither receptor bound to control beads, nor beads coupled at the 8-position, indicating that the binding was specific, and implying that the extracellular domains of the receptors retain their ATP-binding capability when solubilized in DDM. It is possible that, when scaled up to a column format, problems with low binding and elution may be resolved, and ATP-coupled sepharose beads may represent a useful affinity purification procedure for P2X receptors, but we decided to proceed with other methods to purify DdP2XA trimers.

### 3.3 Expression and purification of DdP2XA

**Fig. 3:**
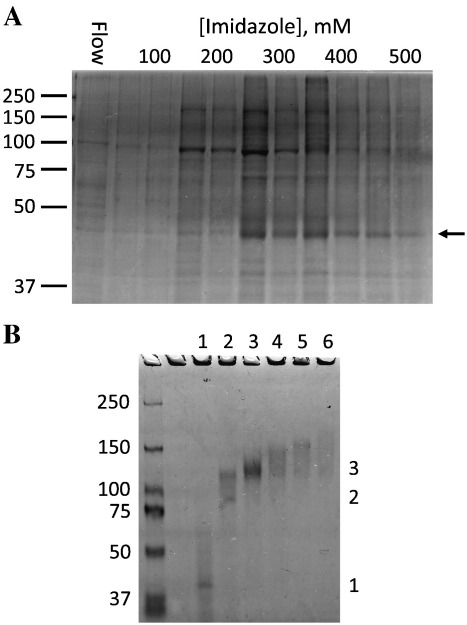
Purification of DdP2XA by affinity chromatography and preparative PFO–PAGE. A: Coomassie-stained gel of the first-stage nickel-sepharose purification of DdP2XA from a 5-litre culture of Sf9 cells. The band corresponding to monomeric DdP2XA, which elutes in the 300–500 mM imidazole fractions, is indicated with an arrow. B: Coomassie-stained PFO–PAGE gel representing pooled fractions (numbers 1–6) from preparative PFO–PAGE. Bands corresponding to monomer (1), dimer (2) and trimer (3) are indicated. Fraction 3 contained pure DdP2XA trimers. Markers in kDa.

Membranes from a 5-litre culture of Sf9 cells expressing DdP2XA were solubilized in DDM and first-stage purification was carried out in batch phase using nickel-sepharose beads to bind the His-tag (Fig. 3A). Partially pure DdP2XA was eluted with increasing concentrations of imidazole; the majority of DdP2XA (major band at 45 kDa on SDS–PAGE; indicated with an arrow) eluted from 300 to 500 mM imidazole. Strong contaminating bands were observed at 100 and 200 kDa; these eluted from 100 to 400 mM imidazole. Preparative PFO–PAGE was then used to purify DdP2XA trimers to homogeneity, using a scaled-up version of the method employed previously to purify hP2X4 from human cells [13]. Samples were separated on an internally cooled tube gel in a Bio-Rad 491 prep-cell. At the bottom of the gel, protein is prevented from diffusing into the running buffer by a chamber bounded by dialysis membrane, and cooled elution buffer flows through this chamber to a fraction collector. Groups of fractions were pooled, concentrated and separated by PFO–PAGE to check the location of the trimer fraction (Fig. 3B). Fraction 1 contained monomeric DdP2XA and other impurities, fraction 2 contained a mixture of dimer and trimer, fraction 3 contained pure trimers, and fractions 4–6 contained high molecular mass impurities. The total yield of pure trimeric DdP2XA was estimated to be approximately 1 mg per 5-litre culture by comparison to known quantities of bovine serum albumin on Coomassie-stained gels.

### 3.4 TEM, single particle analysis and 3D structure of DdP2XA

**Fig. 4:**
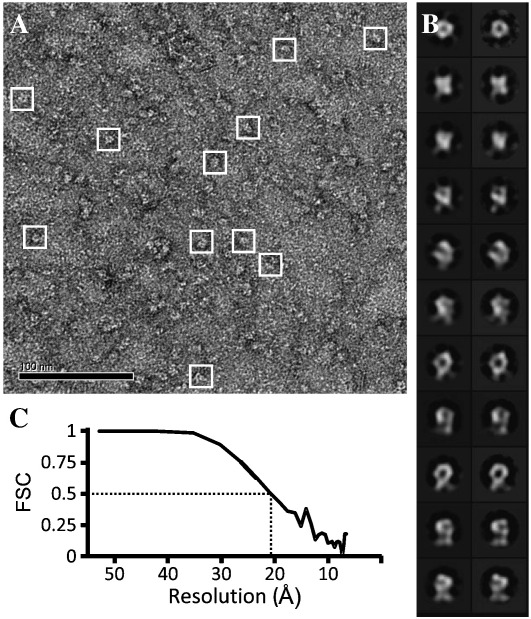
Electron microscopy and single particle analysis of DdP2XA. A: Sample TEM image field (3.30 Å/pixel) of DdP2XA single particles negatively stained with 2%(w/v) uranyl acetate. Single particles are represented in 64 × 64 pixel boxes. B: Selected symmetrized reprojections (left) compared with 2D class averages (right) from the final 3D structure. A good correlation is observed, indicating that the final structure is a fair reflection of the input particles. C: Fourier shell correlation (FSC) analysis of structures of wild-type DdP2XA generated from even- and odd-numbered particles obtained using the command “eotest.” The resolution of the structure given by the FSC value of 0.5 is indicated and corresponds to ~ 21 Å.

**Fig. 5:**
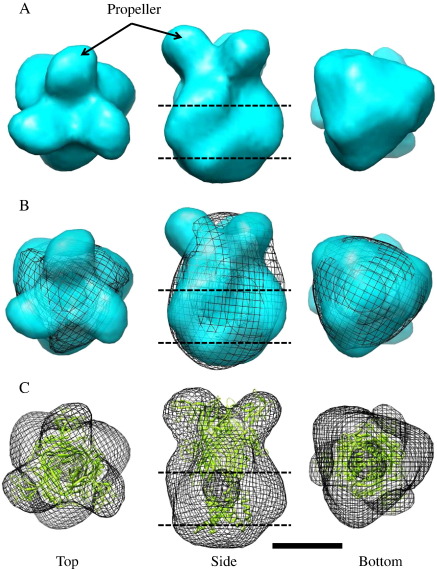
3D structure of Dd2XA derived from single particle analysis. A: Top, side and bottom views of the DdP2XA single particle structure obtained from 5021 single protein particles. The pronounced extracellular domain propeller is indicated with an arrow. B: Top, side and bottom views of the fit of DdP2XA within the previously determined structure of hP2X4 (black mesh) [13], showing the similarity of the overall dimensions. C: Top, side and bottom views of the fit between DdP2XA (black mesh) and the crystal structure of zfP2X4.1 (PDB ID:3I5D; green ribbon). The extracellular domain fit is good but the transmembrane domain fit is poor due to the presence of the DDM micelle in the single particle structure. The approximate position of the transmembrane domain is indicated with dashed lines. Scale bar = 5 nm.

Purified DdP2XA trimers were diluted to a concentration of50 μg/ml and adsorbed on to carbon-coated copper grids for transmission electron microscopy (TEM). A sample TEM image field containing single particles is shown in Fig. 4A. Particles were highly homogeneous and monodisperse, and a total of 5021 particles were selected in 64 × 64 pixel boxes (3.30 Å/pixel) for 3D reconstruction. The correlation between representative 2D class averages and symmetrized reprojections from the final 3D structure (Fig. 4B) was excellent. Fourier shell correlation (FSC) analysis of structures generated from even-and odd-numbered particles using the command “eotest” indicated a resolution of ~ 21 Å (Fig. 4C). The low-resolution 3D structure of DdP2XA displays a prominent extracellular domain propeller (Fig. 5A). The overall dimensions of DdP2XA are similar to those of hP2X4 (Fig. 5B) and the zfP2X4.1 crystal structure (Fig. 5C); however, in DdP2XA the propeller appears to be significantly more pronounced than in hP2X4 (Fig. 5B). When the structure of zfP2X4.1 is fit within the DdP2XA particle structure (Fig. 5C), there is a very good fit with the head domain of the extracellular domain, and a poor fit with the transmembrane domains (expected due to the DDM micelle present in the single particle structure).

### 3.5 N-glycosylation mutagenesis and homology modeling of hP2X4 and DdP2XA

**Fig. 6:**
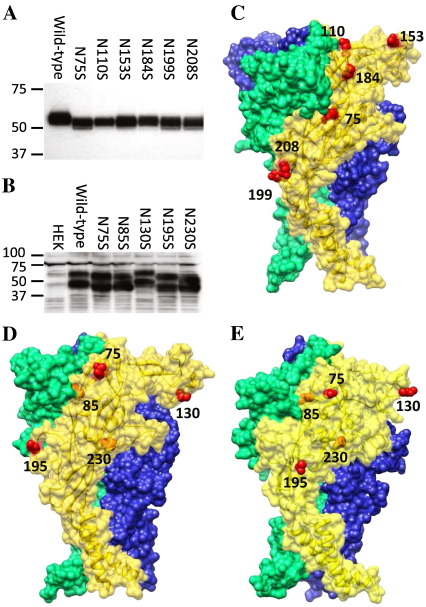
N-glycosylation mutagenesis and molecular modeling of hP2X4 and DdP2XA. Western blots (anti-His tag) of wild-type and potential N-glycosylation site mutants for hP2X4 (A, 10 μg protein/lane) and DdP2XA (B, 50 μg protein/lane). Protein was derived from expression in HEK-293 cells. A reduction in apparent molecular mass indicates that the site is normally glycosylated in HEK cells. All 6 potential sites are utilized in hP2X4, whereas 4 of the 5 sites (Asn^75^, Asn^85^, Asn^195^ and Asn^230^) appear to be utilized in DdP2XA. Molecular models of hP2X4 (C) and DdP2XA both containing (D) and lacking (E) the cysteine-rich head domain (from alignments in Supp. Fig. 1) showing the positions of predicted accessible (red spheres) or inaccessible (orange spheres) N-glycan acceptor sites on one subunit (gold). All 6 sites in the model of hP2X4 are surface-accessible; however, only Asn^75^, Asn^130^ and Asn^195^ are surface-accessible in either DdP2XA model. Although Asn^85^ and Asn^230^ appear to be N-glycosylated in HEK cells, they are not accessible at the surface of the molecular models.

To probe the differences that we observed between the low-resolution structures of hP2X4 and DdP2XA, we generated (i) single-point mutants at each of the potential N-glycosylation consensus sequences (Fig. 6A, B), and (ii) molecular models of each receptor based upon the zfP2X4.1 crystal structure (Fig. 6C-E). DdP2XA lacks many of the extracellular domain cysteine residues conserved in mammalian P2X receptors, and it has recently been suggested that the DdP2XA structure may lack the cysteine-rich head domain [3]. Because of this, we prepared two sequence alignments (Supp Fig. S1): one based on a multiple sequence alignment (where the head domain is present), and one based on a manual alignment (where the head domain is absent [3]). From these alignments we constructed two molecular models of DdP2XA: one containing the head domain (Fig. 6D), and one model which lacks it (Fig. 6E). To provide an initial assessment of quality, the molecular models were manually fitted with the single particle structure of DdP2XA (Supp. Fig. S2), and it was found that the model of DdP2XA containing the head domain (Fig. 6D) gave the best fit with the experimental data.

hP2X4 and DdP2XA contain 6 and 5 N-glycosylation consensus sequences respectively (N-{P}-[ST]-{P}, where {P} represents any amino acid except proline, and [ST] represents serine or threonine). Point mutants for hP2X4 and DdP2XA were expressed in human embryonic kidney (HEK) cells and separated by Western blotting. A reduction in apparent molecular mass for a particular point mutant was assumed to mean that this asparagine residue would normally be N-glycosylated in HEK cells. All 6 mutants caused a reduction in the molecular mass of hP2X4 (Fig. 6A), implying that each position is N-glycosylated in HEK cells. This correlates well with the findings of Qureshi et al., who observed six different N-glycosylation states for rat P2X4 upon glycosidase treatment [22]. Additionally, all 6 sites were surface-exposed in the molecular model of hP2X4 based upon the zfP2X4.1 crystal structure (Fig. 6C), suggesting that the model is a good representation of the structure of hP2X4. For DdP2XA, the results were more complex (Fig. 6B). First, due to the relatively low expression of DdP2XA in HEK cells, more protein had to be loaded, which increased the non-specific background (seen in the non-transfected HEK lane). However, in all samples, 2 strong bands were observed. Mutation of N-glycan sites at positions 75, 85, 195 and 230 caused a reduction in mass of both bands, whereas mutation at position 130 caused no change, consistent with 4 of the 5 sites being N-glycosylated. In both molecular models of DdP2XA based upon the zebrafish P2X4.1 crystal structure (Fig. 6D, E), positions 75, 130 and 195 were predicted to be exposed, whereas positions 85 and 230 were predicted to be buried within the structure, and thus not accessible for N-glycosylation. While positions 75 and 195 are both glycosylated and accessible in the model, position 130 is not glycosylated but accessible, and positions 85 and 230 are glycosylated but not accessible. These discrepancies strongly suggest that neither molecular model of DdP2XA is a fair representation of its native structure.

## 4 Discussion

In the present study we have used Sf9 insect cell culture to express full-length wild-type hP2X4 and DdP2XA, measuring trimer formation in detergent and binding to ATP-coupled sepharose beads. We found that DdP2XA forms stable trimers in DDM, and purified DdP2XA trimers to homogeneity using preparative PFO–PAGE, obtaining a yield of approximately 1 mg pure trimers from a 5-litre culture. Using TEM and single particle analysis, we were able to determine the 3D structure of DdP2XA at a resolution of 21 Å. In addition, we have generated molecular models of hP2X4 and DdP2XA based upon the zfP2X4.1 crystal structure, and probed their accuracy using N-glycosylation mutagenesis.

Sf9 cells have been used previously to express several different P2X receptor subtypes, including rat P2X2 [14,15,23,24], rat P2X3 [24] and zfP2X4.1 [6], and thus represent a proven expression system. The apparent molecular mass of both hP2X4 and DdP2XA monomers (55 kDa and 45–50 kDa respectively) was somewhat lower than that obtained upon expression in human cells (60 kDa [13] and 45–55 kDa [5] respectively). This reflected the differential glycosylation conferred by the different expression systems, because treatment of both human- and insect cell-derived hP2X4 with PNGaseF reduced both bands to the same molecular mass (data not shown).

For hP2X4, the degree of trimer formation for protein produced in Sf9 cells was virtually zero in any detergent tested, which was markedly different to the figure of approximately 50% trimer for DDM-solubilized hP2X4 derived from stably transfected human cells [13]. It is possible that this was due to a high degree of over-expression in the insect cell system leading to the accumulation of incorrectly processed hP2X4 monomers in intracellular organelles, and that, in stably transfected human cells, the lower over-expression level gives the protein more time to be processed correctly. Alternatively, it may be due to incompatibility between the human protein sequence and the insect expression system. It may be possible to use mutagenesis at the subunit interfaces of hP2X4 to strengthen the trimer without compromising channel function; if not, then mammalian expression systems represent the best route for production of hP2X4 for structural studies.

For DdP2XA, a large proportion of stable trimers were present in a variety of detergents, and a similar degree of trimer formation has been observed previously for DdP2XA expressed in human cells [5]. The fact that DdP2XA forms stable trimers in detergent makes it an ideal candidate for structure determination. In this work we have determined a low-resolution structure from single protein particles, but efforts to prepare both 2D- and 3D crystals for higher-resolution studies are ongoing.

One of the most interesting findings of this study is that both DDM-solubilized hP2X4 and DdP2XA were capable of binding to ATP-coupled sepharose beads. The ATP binding sites on P2X receptors are located in the extracellular domain at the interface between subunits [6], so our data suggest that, at least, the extracellular domains of the receptor are correctly folded. The binding properties of both receptors were similar, suggesting that ATP occupies the binding site in both receptors in a similar conformation. Both receptors bound to beads coupled at the γ- and 6-amino-position, but not to beads coupled at the 8-position. One way to interpret this result is to suggest that both the γ-phosphate and the 6-amino group of the adenine moiety must be at least partially exposed when ATP is bound to the receptor, whereas the 8-carbon of the adenine moiety is buried deep within the ATP binding site. Therefore an ATP analog with a large substituent coupled at the 8-position would not be able to bind to the receptor. This interpretation is supported by recent data from Jiang et al., who demonstrated that the ATP analog NCS-ATP (which contains a small, thiol-reactive NCS group, coupled at the 8-position) was able to covalently label rat P2X2 cysteine mutants at two positions (N140C and L186C) located deep within the putative ATP binding site [25]. Our finding that hP2X4 (which formed a very low proportion of oligomers in detergent) bound to ATP-coupled beads implies that the presence of a partial binding site may be sufficient to permit ATP binding. These data could be extended in future experiments by performing binding assays using ATP analogs containing substituents in various positions around the molecule; a cross-linking approach such as that employed by Roberts and Evans on P2X1 [26] could also be used.

Using preparative PFO–PAGE, we were able to isolate homogeneous trimers for structural study. The significant advantage of preparative electrophoresis over gel filtration chromatography is that it is possible to purify several milligrams of protein in one step, without having to use large columns. We are unable to state that PFO–PAGE does not adversely affect P2X receptor structure, because we have not yet been able to overcome the challenges associated with assaying the ion channel function of purified receptors. However, it has been shown that, after purification in PFO, the cystic fibrosis transmembrane conductance regulator (CFTR) retains its function, demonstrating that PFO–PAGE does not severely affect the structure of this particular membrane protein [27].

Purified DdP2XA trimers appeared homogeneous and monodisperse under TEM, and we were able to generate a 21 Å-resolution 3D structure, which possessed similar overall dimensions to the previously determined hP2X4 structure [13] (albeit with significantly pronounced extracellular domain propellers) and the zfP2X4.1 crystal structure [6]. The lateral propellers that we observed in the DdP2XA single particle structure fit well with the cysteine-rich head domain of zfP2X4.1. In addition, a molecular model of DdP2XA containing the head domain was a better fit to the single particle structure than a model of DdP2XA which lacked the head domain. These data imply that the structure of DdP2XA does contain head domains, even though it lacks the majority of conserved cysteine residues within this region, and has been suggested to lack this domain entirely [3].

To probe the structures further, we generated N-glycosylation mutants and used them to validate molecular models of hP2X4 and DdP2XA based on the zfP2X4.1 crystal structure. Our data showed that each of the 6 predicted N-glycan sites on hP2X4 was utilized and predicted to be surface-exposed in the molecular model, implying that the structure of hP2X4 is very similar to that of zfP2X4.1. This is to be expected given the high degree of sequence identity between the two receptors (57%). However, for DdP2XA, only 4/5 of the N-glycan sites were used, and only 3/5 were predicted to be accessible in either molecular model. Asn^130^ was not glycosylated, but was predicted to be surface-exposed in the models. Both Asn^85^ and Asn^230^ were glycosylated, but were not predicted to be surface-exposed in the models. These discrepancies strongly imply that the molecular models of DdP2XA based on the zfP2X4.1 crystal structure are inaccurate; this is not surprising given the low degree of amino-acid sequence identity between the two receptors (16%), and the fact that the accuracy of homology modeling is dependent on sequence identity. The discrepancies in N-glycosylation and the predictions of the molecular model strongly suggest that the structure of DdP2XA is significantly different to that of P2X receptors from higher eukaryotes. Higher-resolution structural data will be required to confirm whether or not this is the case, but it is worth noting the potential errors that could be made by over-interpretation of molecular models derived from sequence alignments which display poor homology.

In summary, we have exploited the Sf9 insect cell system to express hP2X4, and express and purify DdP2XA for direct structural study. We have developed a straightforward pull-down assay to test ATP binding capability, and used N-glycosylation mutagenesis both to confirm the extent of N-linked glycosylation on hP2X4 and DdP2XA and validate homology models of the two receptors. Our work contributes to the understanding of the factors necessary for the successful expression and purification of P2X receptors, highlights the need for construct modification to stabilize trimers (in the case of hP2X4) and demonstrates that DdP2XA is both an interesting and ideal candidate with which to pursue higher-resolution structural studies.
