# Supplementary material for: Expression, purification, electron microscopy, N-glycosylation mutagenesis and molecular modeling of human P2X4 and Dictyostelium discoideum P2XA
Source: Biochim Biophys Acta. 2011 Dec;1808(12):2859–66. doi: 10.1016/j.bbamem.2011.08.025 (PMC3199733; doi:10.1016/j.bbamem.2011.08.025)
Supplement: Supplementary file 1 — Supplementary material. [file mmc1.doc]

**Supplementary Data**

**Expression, purification, electron microscopy, N-glycosylation mutagenesis and molecular modelling of human P2X4 and *Dictyostelium discoideum* P2XA**

Maria Valente, Summer J. Watterson, Mark D. Parker, Robert C. Ford and Mark T. Young

**Supp. Fig. S1.** Sequence alignments of zfP2X4.1 and DdP2XA used for molecular modelling. Alignments of sequence from the ΔzfP2X4-B crystal structure [6] and DdP2XA produced using either a ClustalW multiple alignment of all known full-length P2X receptor sequences (A), or a manual alignment where DdP2XA lacks the cysteine-rich head domain of the receptor (B) [3]. The ΔzfP2X4-B sequence contains the point mutations C51F, N78K and N187R, and lacks residue Arg^137^ due to a chain-break introduced because of poor main-chain electron density in the crystal structure [6]. Identical amino acids are coloured red, and similar residues are coloured blue (similar are IVLM, FYWH, KRH, EQDN). The percentage identities between the two sequences are 18% and 16%, and the percentage similarities are 30% and 25%, for A and B respectively.

**Supp. Fig. S1.**

A. Clustal alignment from multiple sequences

zfP2X4 GTLNRFTQALVIAYVIGYVFVYNKGYQDTDTVLSSVTTKVKG--IALTKT 79

DdP2XA GILHLSFLVGIVAYIVVYSAIIKKGYLFTEVPIGSVRTSLKGPNTFASNL 76

zfP2X4 SELGERIWDVADYIIPPQEDGSFFVLTNMIITTNQTQSKCAENPTPASTC 129

DdP2XA TYCSNQQHNGSTYPFTPLECNYWDEQLALFPVGQDSTFTCTTR---VRLS 123

zfP2X4 TSHRDCKGFNDARGDGVRTGRCVSYSASVKTCEVLSWCPLEKIVDPPNPP 180

DdP2XA KQEANCN-FTDP------TCKFVDEPGSAKNIYIADIESFTILIDHTMYA 166

zfP2X4 LLADAERFTVLIKNNIRYPKFNFNKRNILPNINSSYLTHCVFSRKTDPDC 230

DdP2XA SSSGSQFNAVDLHG----YILNQDGDEVQIDANG---TSIGVSGKPD--- 206

zfP2X4 PIFRLGDIVGEAEEDFQIMAVRGGVMGVQIRWDCDLDMPQSWCVPRYTFR 280

DdP2XA -IMTIGQLLSFGGVSLDQASPVD--SNVSIRYDGVVLFVFITYSNTYTYS 253

zfP2X4 RLDNKDPDNNVAPGYNFRFAKYYKNSDGTETRTLIKGYGIRFDVMVFGQA 330

DdP2XA TSDFKYVYSVQQIANTIYDVPETIILESIHSRLLYKRHGIRVIFIQTGTI 303

zfP2X4 GKFNIIPTLLNIGAGLALLGLVNVICDWIVL 361

DdP2XA GSFHFQTLLLTLVSGLGLLAVATTVVDQLAI 334

B. Manual alignment (based on reference 3)

zfP2X4 GTLNRFTQALVIAYVIGYVFVYNKGYQDTDTVLSSVTTKVKG-------I 74

DdP2XA GILHLSFLVGIVAYIVVYSAIIKKGYLFTEVPIGSVRTSLKGPNTFASNL 76

zfP2X4 ALTKTSELGERIWDVADYIIPPQEDGSFFVLTNMIITTNQTQS-KC--AE 121

DdP2XA TYCS-----NQQHNGSTYPFTPLECNY-WDEQLALFPVGQDSTFTCTTRV 120

zfP2X4 NP-TPASTCTSHR-DCKG-FNDARGDGVRTGRCVSYSASVKTCEVLSWCP 169

DdP2XA RLSKQEANCNFTDPTCKFVDEPGSAKN----------------------- 147

zfP2X4 LEKIVDPPNPPLLADAERFTVLIKNNIRYPKFNFNKRNILPNINSSY-LT 218

DdP2XA ----------IYIADIESFTILIDHTMYASSSGSQFNAVDL-HGYILNQD 186

zfP2X4 HCVFSRKT--DPDCPIFRLGDIVGEAEED-----FQIMAVRGGVMGVQIR 261

DdP2XA GDEVQIDANGTSIGVSGKPDIMTIGQLLSFGGVSLDQASPVDSNVSIR-- 234

zfP2X4 WDCDLDMPQSWCVPRYTFRRLDNK-DPDN-NVAPGYNFRFAKYYKNSDGT 309

DdP2XA YDGVVLFVFITYSNTYTYSTSDFKYVYSVQ---QIANTIYDVPETIILES 281

zfP2X4 ET-RTLIKGYGIRFDVMVFGQAGKFNIIPTLLNIGAGLALLGLVNVICDW 358

DdP2XA IHSRLLYKRHGIRVIFIQTGTIGSFHFQTLLLTLVSGLGLLAVATTVVDQ 331

zfP2X4 IVL 361

DdP2XA LAI 334

**Supp. Fig. S2**

**Supp. Fig. S2.** Fitting of DdP2XA molecular models to the DdP2XA single particle structure. Top and side views showing manual fits of the DdP2XA models derived from the sequence alignments in Supp. Fig. 1, either from the ClustalW alignment (A), or from the manual alignment which omits the head domain (B). The prominent extracellular domain propellers (which fit with the cysteine-rich head domain of the zfP2X4.1 crystal structure (Fig. 5C) are indicated with red circles. It is clear that the fit of density within the extracellular domain propellers is better in model A than in model B, implying that DdP2XA does possess a head domain, even though it lacks many of the conserved cysteine residues in this region [3]. Scale bar = 5 nm.
